# Prevalence and Antifungal Susceptibility Profile of Clinically Relevant *Candida* Species in Postmenopausal Women with Diabetes

**DOI:** 10.1155/2020/7042490

**Published:** 2020-11-26

**Authors:** Sarah Al Halteet, Ahmed Abdel-Hadi, Mohamed Hassan, Mohamed Awad

**Affiliations:** ^1^Department of Biology, College of Science, Taif University, P.O. Box 11099, Taif 21944, Saudi Arabia; ^2^Department of Botany and Microbiology, Faculty of Science, Al-Azhar University, Assiut, Egypt; ^3^Department of Medical Laboratory Sciences, College of Applied Medical Sciences, Majmaah University, Majmaah 11952, Saudi Arabia; ^4^Department of Genetics, Faculty of Agriculture, Menoufia University, 33516 Menoufia, Egypt

## Abstract

The incidence of diabetes mellitus has increased in Saudi Arabia, which has raised the risk of vulvovaginal candidiasis (VVC). This study highlights the prevalence and antifungal susceptibility of *Candida* species among postmenopausal women with diabetes with symptoms of VVC in Taif, a city in Saudi Arabia. Several diagnostic tools were used to differentiate the yeast isolates, including microscopic examination, culture morphology on CHROM agar, further confirmation with the VITEK 2 system, and ITS1 and ITS4 region sequencing. Antifungal susceptibility of the selected *Candida* species was determined using the VITEK 2 system (bioMérieux Inc., USA). Out of the 550 high vaginal swabs investigated, 86 specimens were *Candida* species positive (15.6%) with a significant difference according to age; the positivity in the 45–50 years' age group (12%) was higher than that in the 51–55 years' age group (3.6%). *Candida albicans* was the most common causative agent in 51 samples (59.3%), followed by *C. glabrata* in 21 samples (24.41%) and *C. krusei* in 14 samples (16.27%), with no significant differences between the age groups. Three isolates, including two *C. albicans* and one *C. krusei*, exhibited resistance against all the tested antifungal agents. CHROM agar and VITEK 2 were accurate phenotypic tools to identify *Candida* species with 100% sensitivity and specificity and were consistent with the phylogenetic characterization. The data emphasized the importance of identifying *Candida* species and their antifungal susceptibility among postmenopausal women with diabetes, highlighting the potential risk posed by diabetes in this age group.

## 1. Introduction

Vulvovaginal candidiasis (VVC) is characterized as an overgrowth of opportunistic *Candida* species, particularly *Candida albicans*, inside the vaginal mucosa [1]. An average of 75% of all women experience VVC at least once at some stage in their lifetime [2]. The recurrence rate of VVC is approximately between 40% and 50%, and approximately 5%–10% of such patients develop four or more acute vaginal candidiasis events over a 12-month period [3]. *C. albicans* is the primary etiological agent of VVC, although cases attributable to other nonalbicans *Candida* species are on the rise in immunocompromised women [4]. Some of the most prevalent nonalbicans species are *C. glabrata*, *C. krusei*, *C. parapsilosis*, and *C. tropicalis* [5].

The prevalence of VVC in postmenopausal women is significantly low (ranging from 3% to 7%) among those not receiving hormonal replacement therapy [6, 7]. Diabetes mellitus is a significant risk factor for VVC [5]. Vaginal colonization with *C. albicans* is significantly correlated with glycosuria in women with uncontrolled diabetes and elevated glucose levels in vaginal secretions that develop symptomatic vaginitis [8, 9].

While antifungal agents are routinely used to treat *Candida*-infected women with diabetes without conducting a vaginal or swab test, the early and reliable diagnosis of species-level clinical yeast pathogens is crucial in selecting an effective treatment. In addition, postmenopause lasts nearly one-third of the life spans of women; thus, it is essential to consider changes in the flora of the vagina, the increased risk of VVC, and related health problems in women with diabetes. The number of women with diabetes in Saudi Arabia is increasing, requiring more studies for this group. Therefore, the current study is aimed at using phenotypic and genotypic tools to identify *Candida* species in high vaginal swab samples collected from postmenopausal women with diabetes with symptoms of VVC.

## 2. Materials and Methods

### 2.1. Sample Collection

This study was conducted at King Faisal Specialist Hospitals in Taif, a city in the Mecca Province of Saudi Arabia. A total of 550 high vaginal swab specimens were collected from consenting postmenopausal women with diabetes aged 45–55 years with symptoms of VVC, such as vaginal itching, burning sensation upon urination, and vaginal discharge with bad odor, between September 2017 and April 2018. The gynecologist collected samples after speculum examination with sterile cotton-tipped swabs. The collected swabs were placed in sterile tubes containing 1 mL of sterile 9% saline solution and transferred to the Microbiology Laboratory, Department of Biology, Faculty of Science, Taif University, Saudi Arabia. The Ministry of Health Ethical Committee approved the study (ethical approval number 04).

### 2.2. Direct Examination of Specimens

The collected specimens were examined under a microscope, as described by Suhonen et al. [10]. Briefly, 10% KOH was added to the specimens, followed by incubation for 5 to 10 min. Next, 1 mL glycerol (0.25%) was added to maintain the moistness of the specimens. The KOH-digested specimens were coverslipped before microscopic examination.

### 2.3. Growth on Culture Media

The collected vaginal swab specimens were inoculated on Sabouraud dextrose agar (SDA) with 0.01% chloramphenicol. All plates were incubated at 37°C for 7 days with daily assessment of growth. The purified growing colonies and macroscopic characteristics were determined. The isolated colonies were inoculated on chromogenic medium (CHROM Agar Candida®) and incubated for 72 h at 37°C. Samples were analyzed according to coloration and colony morphology [11].

### 2.4. VITEK 2 Compact System

*Candida* species were identified with the VITEK 2 compact system (bioMérieux Inc., USA) in Medical Laboratories Science Department, College of Applied Medical Sciences, Majmaah University, Saudi Arabia, using YST ID REF21343 (yeast identification) test cards. The test procedures were performed according to the manufacturer's instructions. *Candida* suspensions for VITEK 2 analysis were prepared by mixing the colony with 3 mL of 0.45% sterile saline to obtain turbidity equivalent to that of 0.5 McFarland standard using a DensiChek colorimeter (bioMérieux). The test cards were automatically loaded with the suspensions, sealed, and incubated for 18 h at 35.5°C using the VITEK 2 instrument. The identification of *Candida* species was achieved by reading cards and comparing with the database using software version 07.01.

To determine the antifungal susceptibility of 24 selected *Candida* species, 145 *μ*L of the fungal suspension was drawn into 3 mL of 0.45% saline solution to adjust the fungal cell density. VITEK cards were inoculated with the suspension vials and loaded into the VITEK 2 automated reader-incubator using AST-YS01 cards. Results were interpreted using the VITEK 2 Compact software version 07.01.

### 2.5. DNA Sequencing

Genomic DNA was extracted from purified yeast cells using the method described by Looke et al. [12] with some modifications. The universal primers, ITS1 (5′-TCCGTAGGTGAACCTGCG-3′) and ITS4 (5′-TCCTCCGCTTATTGATATGC-3′), were used to amplify the ITS regions of the selected species [13]. PCR amplification was conducted in a total reaction mixture volume of 25 mL using 1x PCR buffer (DreamTaq™) in a C1000TM Thermo Cycler (Bio-Rad, Germany). PCR products were purified using QIAquick purification kits (QIAGEN, Valencia, CA, USA) according to the manufacturer's instructions. The DNA amplicons were sequenced using the Gene Analyzer 3121 sequencer with the same primers ITS1 and ITS4 (Macrogen Co., Seoul, South Korea). The ITS sequences were analyzed using BioEdit version 7.2.5. A total of 32 isolates, including 18 *C. albicans*, 11 non-*Candida* species, and 3 *Saccharomyces cerevisiae*, were examined. Isolates were identified by comparing the sequencing data against databases using the BLAST of the GenBank database (http://www.ncbi.nlm.nih.gov/BLAST/).  Table 1 shows the GenBank accession numbers of the ITS1 and ITS4 regions of type (or reference) isolates of the 32 species sequenced.

### 2.6. Statistical Analysis

Statistical analysis was performed using SPSS statistical software. The correlation analysis between the variables (age, *Candida* prevalence, and *Candida* species) was performed separately with the response variable using a one-way analysis of variance. A *P* value less than 0.05 was considered statistically significant.

## 3. Results

### 3.1. Microscopic Characterization

Direct microscopic examination with 10% KOH-treated 550 vaginal swab samples collected from postmenopausal women with diabetes with VVC symptoms showed that 70 specimens (12.7%) had yeast cells with or without pseudohyphae, 439 samples (79.8%) had bacterial cells with or without yeast cells, and 41 samples (7.5%) had no noteworthy observation.

### 3.2. Macroscopic Characterization on CHROM Agar Medium

**Figure 1:**
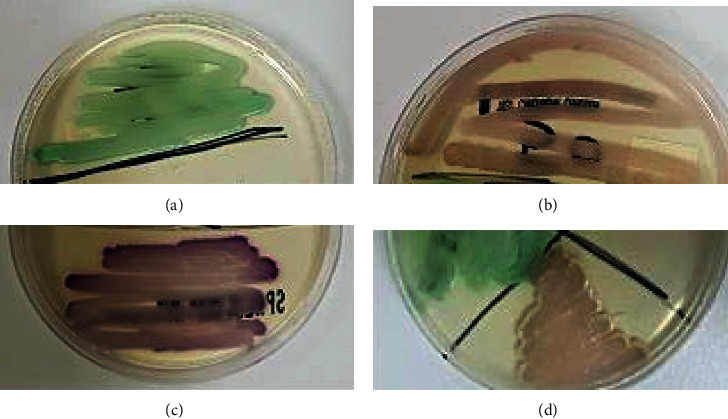
Appearance of yeast colonies on CHROM Agar Candida: (a) *Candida albicans*, (b) *Saccharomyces cerevisiae*, (c) *Candida krusei*, and (d) *Candida glabrata*.

The swab samples (*n* = 550) were cultured on SDA for 7 days. A total of 96 samples (17.5%) were positive for culture. The following morphological characteristics of the isolated colonies were observed: size, small to large; color, white to creamy; shape, round or curved; texture, smooth and soft to wrinkled; and odor, characteristic of yeast. Chromogenic medium is capable of distinguishing between *C. albicans* and nonalbicans *Candida* species, depending on color. Of the 96 isolates grown on SDA, 86 isolates showed the characteristics of *Candida* species, with a specific color on CHROM agar after incubation for 48 h. A total of 51 isolates were classified as *C. albicans* colonies, where the color was light to dark green; 21 isolates were classified as *C. glabrata*, where the color was white to mauve; 14 isolates were classified as *C. krusei*, where a pinkish color had developed; and 10 isolates were classified as *S. cerevisiae*, where a brown color had developed (Figure 1).

### 3.3. VITEK 2 System Characterization

**Figure 2:**
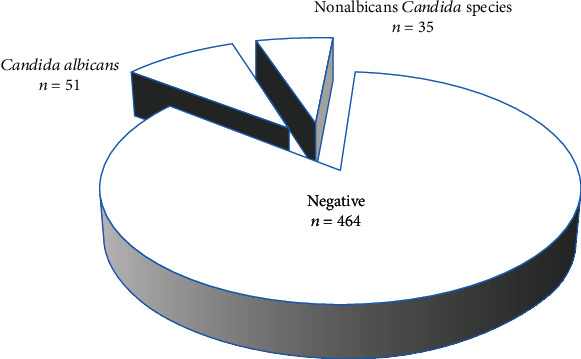
Frequency of *Candida albicans* and nonalbicans *Candida* species isolated from postmenopausal women with diabetes with vulvovaginal candidiasis based on VITEK 2 characterization.

We validated 86 isolates showing the color characteristic of *Candida* species on CHROM agar with the VITEK 2 compact system (Table 2). A total of 51 isolates were identified as *C. albicans* (59.3%), 21 were *C. glabrata* (24.41%), and 14 were *C. krusei* (16.27%), with no significant differences between the age groups (Figure 2).

### 3.4. Phylogenetic Characterization

**Figure 3:**
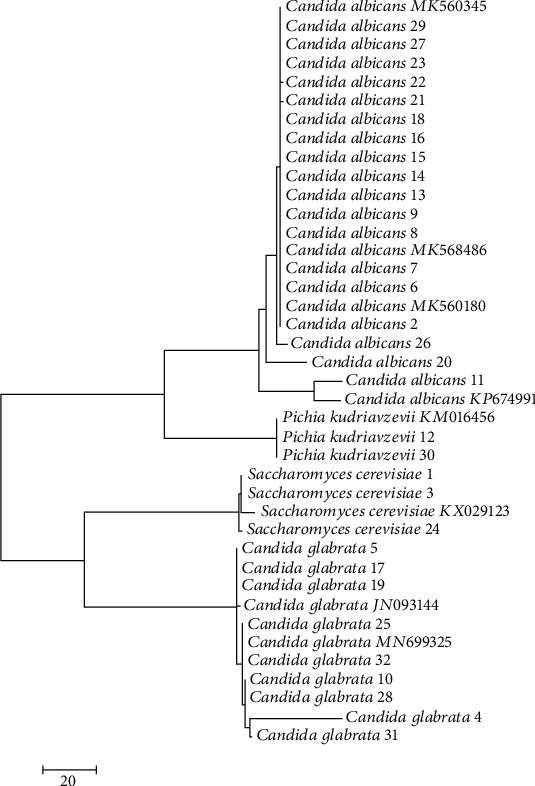
Neighbor-joining tree showing genetic diversity of *Candida* isolates based on the sequence analysis of the ITS region.

Twenty-nine isolates of *Candida* species identified using VITEK 2 (bioMérieux) and three *S. cerevisiae* isolates identified by CHROM agar were further subjected to molecular typing based on ITS region 1 and 4 genes to define the genetic similarities among the tested isolates. A phylogenetic tree was constructed based on ITS region sequence analysis using the neighbor-joining tool in the MEGA 7.1 program. Bootstrap analysis of the ITS region with 500 bootstrap replications demonstrated two main clusters (Figure 3). Most isolates of *Candida* species were included in the first main cluster, which was supported with a bootstrap value of 100%. The first subclade of the first main cluster contained *C. albicans* C-2, C-8, C-9, C-11, C-13, C-14, C-16, C-20, and C-21, which were closely similar to *C. albicans* MK560345 and *C. albicans* KP674991, with similarity ranging from 94% to 98%. The second subclade contained *C. albicans* C-6, C-7, C-18, C-22, C-23, C-26, C-27, and C-29, which were closely similar to *C. albicans* MK568486 and *C. albicans* MK580180, with similarity ranging from 93% to 100%. The third subclade contained *Pichia kudriavzevii* C-12 and C-30, which were closely similar to *P. kudriavzevii* KM016456, with similarity ranging from 92% to 98%. The other *Candida* isolates in the first subclade of the second main cluster included C-4, C-5, C-10, C-17, C-19, C-25, C-28, C-31, and C-32, which were closely similar to *C. glabrata* MN699325 and *C. glabrata* JN093144, with similarity ranging from 98% to 100%. Finally, the second subclade of the second main cluster contained *S. cerevisiae* strains C-1, C-3, and C-24, which were closely similar to *S. cerevisiae* KX029123, with similarity ranging from 98% to 100%.

### 3.5. Antifungal Susceptibility Testing

**Figure 4:**
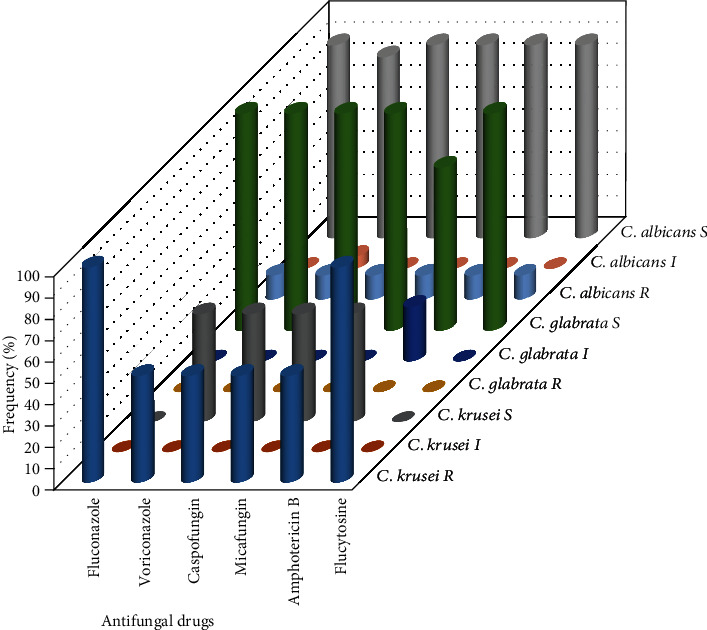
Antifungal susceptibility pattern of *Candida* species associated with postmenopausal women with diabetes (*Candida* *albicans* = 18; *Candida* *glabrata* = 4; *Candida* *krusei* = 2), where S = susceptible, I = intermediate, and R = resistance.

A total of 24 *Candida* species, including 18 *C. albicans*, 4 *C. glabrata*, and 2 *C. krusei*, were examined for their susceptibility to six antifungal agents using the VITEK 2 system. Our antifungal susceptibility results (Table 3) indicated that three *C. glabrata* isolates were sensitive to all tested antifungal agents, except *C. glabrata* MN419362, whose susceptibility was intermediate to amphotericin B. A total of 2 of 18 *C. albicans* isolates exhibited resistance to all tested antifungal agents. In addition, *C. krusei* MN419370 showed resistance to all tested antifungal agents, while *C. krusei* MN419388 was resistant only to fluconazole and flucytosine (Figure 4).

## 4. Discussion

Data on the prevalence of VVC in women with diabetes in Saudi Arabia are insufficient. Regrettably, VVC is not a notifiable disease and is commonly treated based on symptoms and signs without laboratory diagnosis. Accurate identification of the etiological agent of VVC is important for the management of empirical antifungal therapy [14]. They noted that the incidence of errors in the diagnosis of VVC by physicians based on clinical evidence alone was high. Consequently, the variety of yeasts responsible for causing VVC and the profile of their drug susceptibility have not been identified in Saudi Arabia.

According to the World Health Organization reports, the prevalence of diabetes mellitus in Saudi Arabia is seventh globally and second in the Middle East [15]. One risk factor that increases the incidence of diabetes mellitus in Saudi Arabia has been reported to be an increase in age [16]. It has been found that the average age of patients with diabetes is 55.3 years and that females under the age of 50 years have a higher prevalence of this disease than males of the same age. Women with diabetes are the most vulnerable to VVC, including those caused by *Candida* species [17]. This may be due to decreased immune response, frequency, type of diabetes, and glucose regulation [18].

**Figure 5:**
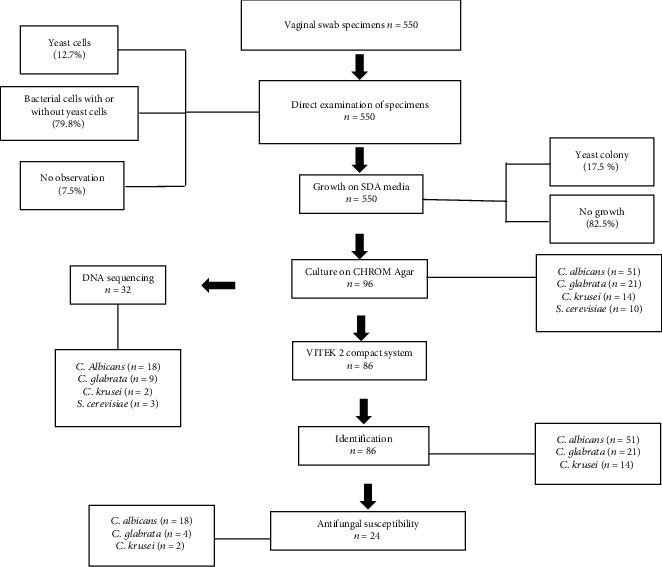
Flow diagram for the identification of *Candida* species associated with postmenopausal women with diabetes.

In the current study, the initial identification of *Candida* isolates was based on microscopic examination, colony morphology on CHROM agar, and further confirmation with VITEK 2. ITS regions of 29 *Candida* clinical isolates were subjected to PCR amplification using ITS 1 and ITS 4 primers and subsequently sequenced (Figure 5). The overall sensitivity and accuracy of direct microscopy to predict VVC were 81.3% and 100%, respectively. Previously, it has been reported that negative smear results do not preclude the presence of disease, and a 10 min delay in examination of the smear may decrease the sensitivity to 20% [19].

Interestingly, the CHROM agar results were consistent with those of VITEK 2 that showed 100% sensitivity and accuracy. A previous study evaluated the performance of 521 yeast strains, including 23 species of chromogenic *Candida* [20]. Their findings were similar to those of the current study, where the sensitivity and accuracy of the chromogenic medium were both more than 99.4% for each species. Another study by Melhem et al. [21] examined the VITEK 2 system to identify 11 quality control strains and 32 clinically relevant yeast strains. They reported that the VITEK 2 system identified all the challenged strains with 100% sensitivity and accuracy. The presented results show that some conventional techniques, such as CHROM agar, are still valid for diagnosing VVC and are consistent with VITEK 2 results and molecular tools to identify *Candida* species. Previously, it has been documented that the identification of clinically important yeasts by sequencing of ITS regions is an accurate method for species-level identification [22, 23].

Our results showed that the prevalence of VVC in symptomatic postmenopausal women with diabetes was 15.6% (86/550) with a significant difference according to age; VVC prevalence was higher in patients aged 45–50 years (12%) than in those aged 51–55 years (3.6%). Our results are similar to those of Gunther et al. [24], who reported that the prevalence of VVC in Brazil was 18.8% in women with diabetes and 11.8% in women without diabetes in the control group. This is in contrast to other studies that have reported that postmenopausal women rarely experience VVC [25, 26]. This may discuss the influence of diabetes on the increase in the incidence of VVC in postmenopausal women. The current study showed that *C. albicans* (59.3%) was the most common causative agent, followed by *C. glabrata* (24.41%) and *C. krusei* (16.27%), with no significant differences between ages. Similarly, Sherry et al. [27] have reported that the most common causative agent of VVC is *C. albicans*, appearing in more than 90% of infections, but there is an increase in the prevalence of nonalbicans *Candida* species based on the geographical location. In contrast, Goswami et al. [8] showed that *C. glabrata* is the most predominant *Candida* species (39%) in India isolated from women with diabetes with VVC. In a study comprising 111 consecutive female patients with diabetes with VVC, Ray et al. [28] documented that *C. glabrata* was isolated from 68 (61.3%) and *C. albicans* from 32 (28.8%) patients.

The susceptibility profiles of *Candida* species isolated from postmenopausal women with diabetes were not the same, and some species have been shown to acquire resistance. Currently, two major groups of antifungal drugs, polyene antifungal drugs and pyrrole ring drugs, are predominantly utilized for the treatment of VVC in clinical practice. Polyene antifungal drugs, including amphotericin B, exert a high antifungal effect but are markedly toxic [29]. Pyrrole ring antifungal drugs, including azoles, such as fluconazole, are linked to inhibiting the synthesis of ergosterol in fungi, thus destroying the integrity of the fungal cell membrane and exerting antifungal effects [30]. A single dose of fluconazole is widely used in patients without diabetes but with symptomatic VVC because of its effectiveness and efficient dosing schedule [31]. In our study, the resistance levels of *C. albicans*, *C. glabrata*, and *C. krusei* to fluconazole were 11.1% (2/18), 0% (0/4), and 100% (2/2), respectively, indicating that *C. glabrata* is more susceptible to fluconazole than other species. In contrast to our results, Goswami et al. [9] reported that the most common causative agent of VVC in women with diabetes was *C. glabrata*, showing a frequency of 54.1% and exhibiting resistance to fluconazole treatment in 67.1% of the patients. Interestingly, four *Candida* species (two *C. albicans* and two *C. krusei*) showed marked resistance to most of the antifungal agents tested. This could be due to many factors, including previous exposure to antifungal drugs, the development of resistance genes, improvements in membrane lipid fluidity and asymmetry, the involvement of other chemotherapeutic drugs, and the inherent resistance of *Candida* species.

## 5. Conclusions

To the best our knowledge, this is the first study to reveal the prevalence of *Candida* species in postmenopausal women with diabetes in Saudi Arabia. Diabetes mellitus is a risk factor for *Candida* colonization in postmenopausal women throughout one-third of their life spans. Based on the data presented, a direct smear and high vaginal swab culture should be validated using appropriate methods such as the VITEK 2 system and *Candida* molecular identification technique at the species level. Additional studies at different locations in Saudi Arabia are required to establish strategies to avoid the inherent risk of developing VVC as well as related health issues within this group. Our results indicate that the susceptibility profiles for *Candida* species are not the same. Increased use of antifungals should be regulated through the current national surveillance program. Our study has a limitation that the control samples were not planned, as the study was initiated with a small number of samples. However, a larger study is planned in the near future to overcome the aforementioned limitation.

## Figures and Tables

**Table 1:** Molecular identification of *Candida* species isolated from postmenopausal women with diabetes based on rDNA sequencing and matching with the NCBI GenBank database.

Strain number	Proposed taxa	BLAST accession number	Query cover (%)	Identity (%)	Strain accession number
C-1	*Saccharomyces cerevisiae*	JN093144.1	92	95	MN419359
C-2	*Candida albicans*	MK560345.1	98	98	MN419360
C-3	*Saccharomyces cerevisiae*	KX029123	95	96	MN419361
C-4	*Candida glabrata*	JN093144	95	96	MN419362
C-5	*Candida glabrata*	JN093144	93	92	MN419363
C-6	*Candida albicans*	MK560180.1	96	99	MN419364
C-7	*Candida albicans*	MK560180.1	98	100	MN419365
C-8	*Candida albicans*	MK568486.1	92	99	MN419366
C-9	*Candida albicans*	MK568486.1	97	98	MN419367
C-10	*Candida glabrata*	JN093144	100	98	MN419368
C-11	*Candida albicans*	KP674991.1	93	92	MN419369
C-12	*Pichia kudriavzevii*	KM016456	90	92	MN419370
C-13	*Candida albicans*	KP674991.1	97	98	MN419371
C-14	*Candida albicans*	MK568486.1	98	98	MN419372
C-15	*Candida albicans*	MK568486.1	95	99	MN419373
C-16	*Candida albicans*	MK568486.1	99	100	MN419374
C-17	*Candida glabrata*	JN093144	92	95	MN419375
C-18	*Candida albicans*	KP674991.1	99	100	MN419376
C-19	*Candida glabrata*	JN093144	96	97	MN419377
C-20	*Candida albicans*	KP674991.1	98	98	MN419378
C-21	*Candida albicans*	KP674991.1	97	99	MN419389
C-22	*Candida albicans*	MK560345.1	100	99	MN419380
C-23	*Candida albicans*	MK568486.1	98	100	MN419381
C-24	*Saccharomyces cerevisiae*	KX029123	97	98	MN419382
C-25	*Candida glabrata*	JN093144	99	99	MN419383
C-26	*Candida albicans*	KP674991.1	98	97	MN419384
C-27	*Candida albicans*	MK560345.1	97	99	MN419385
C-28	*Candida glabrata*	JN093144	97	98	MN419386
C-29	*Candida albicans*	MK568486.1	99	100	MN419387
C-30	*Pichia kudriavzevii*	KM016456	95	96	MN419388
C-31	*Candida glabrata*	JN093144	96	97	MN419389
C-32	*Candida glabrata*	JN093144	96	97	MN419390

**Table 2:** Proportion of *Candida* species isolated from postmenopausal women with diabetes with vulvovaginal candidiasis.

*Candida* spp.	Mean age of patients	Number of isolates (*n* = 86)	Percentage (%)
*C. albicans*	49.58 ± 3.11	51	59.3
*C. glabrata*	50.47 ± 2.54	21	24.4
*C. krusei*	48.50 ± 1.79	14	16.3

**Table 3:** Antifungal susceptibility of the selected *Candida* species associated with postmenopausal women with diabetes.

Strains	Fluconazole		Voriconazole		Caspofungin		Micafungin		Amphotericin B		Flucytosine	
	MIC	Interp.	MIC	Interp.	MIC	Interp.	MIC	Interp.	MIC	Interp.	MIC	Interp.
*Candida albicans* MN419360	≤1	S	≤0.12	S	≤0.25	S	≤0.06	S	0.5	S	≤1	S
*Candida albicans* MN419364	≤1	S	≤0.12	S	≤0.25	S	≤0.06	S	0.5	S	≤1	S
*Candida albicans* MN419365	≤1	S	≤0.12	S	≤0.25	S	≤0.06	S	0.5	S	≤1	S
*Candida albicans* MN419366	≤1	S	≤0.12	S	≤0.25	S	≤0.06	S	0.5	S	≤1	S
*Candida albicans* MN419367	≥64	R	≥ 8	R	≥4	R	≥4	R	≥16	R	32	R
*Candida albicans* MN419369	≤1	S	≤0.12	S	≤0.25	S	≤0.06	S	0.5	S	≤1	S
*Candida albicans* MN419371	≤1	S	≤0.12	S	≤0.25	S	≤0.06	S	0.5	S	≤1	S
*Candida albicans* MN419372	≥64	R	≥8	R	≥4	R	≥4	R	8	R	32	R
*Candida albicans* MN419373	≤1	S	≤0.12	S	≤0.25	S	≤0.06	S	0.5	S	≤1	S
*Candida albicans* MN419374	≤1	S	2	I	≤0.25	S	≤0.06	S	1	S	≤1	S
*Candida albicans* MN419376	≤1	S	≤0.12	S	≤0.25	S	≤0.06	S	0.5	S	≤1	S
*Candida albicans* MN419378	≤1	S	≤0.12	S	≤0.25	S	≤0.06	S	0.5	S	≤1	S
*Candida albicans* MN419389	4	S	≤0.12	S	≤0.25	S	≤0.06	S	1	S	≤1	S
*Candida albicans* MN419380	8	S	≤0.12	S	≤0.25	S	≤0.06	S	1	S	≤1	S
*Candida albicans* MN419381	≤1	S	≤0.12	S	≤0.25	S	≤0.06	S	0.5	S	≤1	S
*Candida albicans* MN419384	≤1	S	≤0.12	S	≤0.25	S	≤0.06	S	0.5	S	≤1	S
*Candida albicans* MN419385	≤1	S	≤0.12	S	≤0.25	S	≤0.06	S	0.5	S	≤1	S
*Candida albicans* MN419387	≤1	S	≤0.12	S	≤0.25	S	≤0.06	S	0.5	S	≤1	S
*Candida glabrata* MN419362	8	S	≤0.12	S	≤0.25	S	≤0.06	S	2	I	≤1	S
*Candida glabrata* MN419363	4	S	≤0.12	S	≤0.25	S	≤0.06	S	≤0.25	S	≤1	S
*Candida glabrata* MN419368	≤1	S	≤0.12	S	≤0.25	S	≤0.06	S	0.5	S	≤1	S
*Candida glabrata* MN419389	≤1	S	≤0.12	S	≤0.25	S	≤0.06	S	0.5	S	≤1	S
*Candida krusei* MN419370	≥64	R	≥8	R	≥4	R	≥4	R	≥16	R	≥64	R
*Candida krusei* MN419388	32	R	≤0.12	S	≤0.25	S	≤0.12	S	0.5	S	8	R

S = susceptible; I = intermediate; R = resistance; MIC = minimum inhibition concentration; Inter = interpretation.

## Data Availability

The data used to support the findings of this study are available from the corresponding author upon request.
